# An Uncommon Incidence of Drug-Induced Immune Hemolytic Anemia Secondary to Ceftriaxone

**DOI:** 10.7759/cureus.20682

**Published:** 2021-12-25

**Authors:** Aishwarya Sharma, Shannon Chamberlain, Devendranath Mannuru, Abhishek Matta

**Affiliations:** 1 Medicine, University of North Dakota School of Medicine and Health Sciences, Grand Forks, USA; 2 Internal Medicine, Sanford Health, Fargo, USA; 3 Internal Medicine, University of North Dakota School of Medicine and Health Sciences, Grand Forks, USA

**Keywords:** antibodies, anemia, cephalosporin, complement, coombs test, immune-mediated hemolysis

## Abstract

A 69-year-old female with a history of psoriatic arthritis was diagnosed with septic arthritis and started on broad-spectrum antibiotics. She underwent left hip excisional debridement of her prosthetic hip joint which grew group B Streptococcus (*S. agalactiae*). She was switched to IV ceftriaxone 2 g daily and her hemoglobin decreased to 5.4 g/dL on day 11. Peripheral blood smear showed normochromic normocytic anemia and thrombocytopenia without the presence of schistocytes. Increased lactate dehydrogenase (LDH), decreased haptoglobin and hemoglobin, and positive direct Coombs test (DCT) led to a presumptive diagnosis of drug-induced immune hemolytic anemia (DIIHA). As a result, she was switched from ceftriaxone to IV ertapenem 500 mg every 24 hours and oral prednisone 60 mg for four days during the initial phase. Her hemoglobin, LDH, and haptoglobin trended towards normal limits, further supporting the diagnosis of DIIHA secondary to ceftriaxone.

## Introduction

Ceftriaxone is commonly used due to its wide coverage of bacterial infections, variable dosing, and limited cross-reactivity with other drugs. It is a third-generation cephalosporin antibiotic that can cause drug-induced immune hemolytic anemia (DIIHA) at an estimated incidence of one per million per year [1,2]. Sequelae include shock, organ ischemia, and renal failure. Laboratory investigations usually identify hemolysis by decreased hemoglobin, low haptoglobin, elevated lactate dehydrogenase (LDH), and hyperbilirubinemia. Direct Coombs test (DCT) is positive for anti-C3 and/or IgG/IgM [3]. Drug-induced antibodies trigger hemolysis and cause a subsequent decrease in hemoglobin levels.

A delay in recognition of DIIHA cases can result in under-diagnosis and poorer outcomes. Immediate discontinuation of ceftriaxone remains the preferred therapy option [2]. Given the diagnostic difficulty associated with DIIHA, it is important to include DIIHA in the differential when there is a decrease in hemoglobin during ceftriaxone therapy. Once a diagnosis has been made, it is important to include ceftriaxone in the patient’s chart to avoid reexposure.

## Case presentation

A 69-year-old female with a history of psoriatic arthritis for which she was on weekly methotrexate 20 mg subcutaneous injections and Remicade 600 mg every eight weeks presented to an outside facility with chills and left hip pain in the setting of her prosthetic hip joint. She was diagnosed with septic arthritis and started on IV vancomycin and IV Zosyn 3.375 g every six hours after undergoing left hip excisional debridement with polyethylene and head exchange on the day of admission. Her blood and operative cultures came back positive for group B Streptococcus (*S. agalactiae*), so her antibiotics were switched to IV ceftriaxone 2 g daily on day three. She also had acute kidney injury likely due to hypotension and blood loss anemia in the immediate post-operative period with creatinine peaking at day seven of the hospital stay with subsequent improvement. The patient received a blood transfusion when her hemoglobin dropped below 7 g/dL. Acute tubular necrosis was not noted on urine analysis.

**Figure 1:**
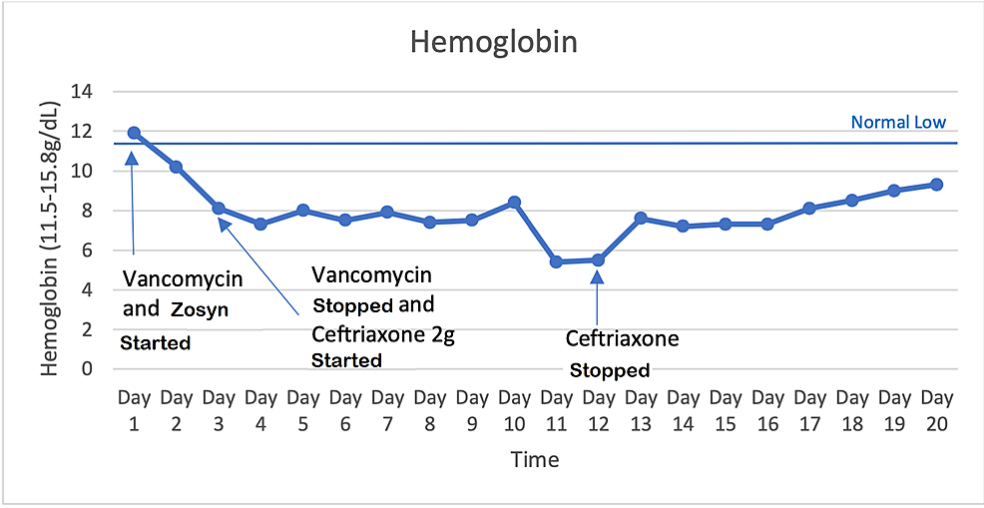
Hemoglobin down trended for nine days after starting ceftriaxone in the outside facility and then rose following discontinuation.

On day 11, her hemoglobin down trended to 5.4 g/dL from 11.9 g/dL, and she was transferred to our facility for further care (Figure 1). She lost 1000 mL of blood during surgery which accounted for the initial drop in hemoglobin. Her vital signs were within normal limits when she arrived at our facility, and her left hip dressing was clean, dry, and intact (Table 1). There was no swelling/hematoma or tenderness to palpation at the surgical site. Her peripheral blood smears showed normochromic normocytic anemia and thrombocytopenia without the presence of schistocytes.

**Table 1:** Vital signs were within normal limits on admission to our facility. SpO_2_: oxygen saturation

Vital Signs	Patient’s Value
Temperature	97.9°F (36.6°C)
Pulse	68 beats per minute
SpO_2_	99% on room air
Blood pressure	138/72 mmHg
Respiratory rate	14 breaths per minute
Weight	112.9 kg (249 lbs)

**Figure 2:**
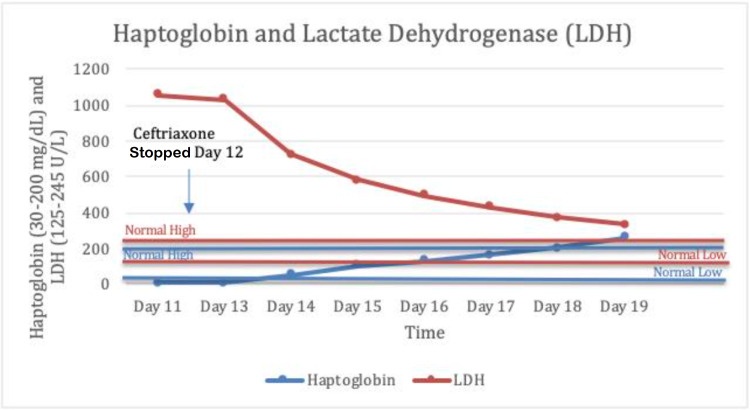
Haptoglobin levels rose and LDH levels decreased following discontinuation of ceftriaxone.

Workup showed increased LDH and decreased haptoglobin, hemoglobin, and platelet count (Figures 1, 2, and Table 2). Subsequently, her direct Coombs test (DCT) came back positive for anti-IgG, C3b antihuman globulin (AHG) (Table 3). A presumptive diagnosis of drug-induced hemolytic anemia (DIIHA) was made given the onset of symptoms and lab findings following ceftriaxone administration.

**Table 2:** Lab results of the patient on admission to our facility. “D1” stands for Day 1, etc., the values in italics indicate abnormal results, and the empty boxes indicate days when labs tests weren’t performed. AST: aspartate aminotransferase; ALT: alanine transaminase; LDH: lactate dehydrogenase; TIBC: total iron-binding capacity

Patient’s Value	D1	D3	D6	D11	D13	D16	D20	Reference Ranges
WBC	10.7	12.3	6.6	9.8	12.6	15.2	9.0	4.0-11.0 K/uL
Hemoglobin	11.9	8.1	7.5	5.4	7.6	7.3	9.3	11.5-15.8 g/dL
Platelet count	268	260	321	143	127	142	161	140-400 K/uL
Total bilirubin	0.8			1.1	1.2	0.5	0.6	0.2-1.2 mg/dL
Creatinine	0.67	3.69	6.65	4.26	3.32	1.83	1.54	0.60-1.10 mg/dL
AST	11			51	25	12	11	0-35 U/L
ALT	8			11	11	10	6	0-55 U/L
Alkaline phosphatase	132			98	165	102	96	30-150 U/L
LDH				1056	1031	499		125-245 U/L
Haptoglobin				8	8	132		30-200 mg/dL
Ferritin			595	1774				5-200 ng/mL
Iron saturation			15	16				20-50%
Iron total			24	41				50-170 ug/dL
TIBC			159	249				250-400 ug/dL
Reticulocyte count				0.05				0.02-0.09 M/uL

**Table 3:** Direct Coombs test results came back positive. DCT: direct Coombs test; DAT: direct antiglobulin testing; AHG: antihuman globulin

Test	Patient’s Value
DCT	DAT reaction strength 3+ anti-IgG, C3b AHG
DCT complement	Negative

The patient did not have any additional bleeding at the surgical site. There were no clinical signs of GI or retroperitoneal bleeds. The low haptoglobin level indicated intravascular hemorrhage. The positive DCT and the improvement in hemoglobin following cessation of ceftriaxone therapy supported our diagnosis.

Therefore, ceftriaxone was discontinued on day 12, and the patient was treated with IV ertapenem 500 mg every 24 hours and oral prednisone 60 mg for four days during the initial phase and then stopped. The patient was also transfused with three units of packed RBCs. Upon recheck on day 13, her hemoglobin had improved to 7.6 g/dL and her platelet count was 104 K/uL (Figure 1).

Her hemoglobin stabilized despite discontinuing steroids, further supporting the diagnosis of DIIHA secondary to ceftriaxone. At the time of discharge, her hemoglobin was 9.3 g/dL, LDH was 334 U/L, and haptoglobin was 260 mg/dL (Figures 1, 2). Her hemoglobin was back in the normal range two months after discharge.

## Discussion

DIIHA is an adverse complication requiring prompt diagnosis and discontinuation of the inciting medication [2-4]. It is important to distinguish the etiology of drug-induced hemolysis between direct binding and lysis of erythrocytes by the drug and a drug-induced immunologic reaction [1,3-6]. Drug-induced immunologic reactions are a type of DIIHA that can be triggered by drug-induced antibodies. These antibodies can be further divided into a drug-dependent mechanism that requires the presence of the drug to trigger the generation of antibodies and subsequent hemolysis or a drug-independent mechanism in which circulating autoantibodies trigger hemolysis in the absence of the drug [1-3,5,6]. Ceftriaxone has been shown to induce IgM and IgG antibodies which activate complement and trigger intravascular hemolysis by mounting an immune complex response [1-3]. As a result, the DCT is usually C3 and IgG positive [1-3,7]. Our patient had a positive DCT for IgG and C3b. Large amounts of hemolysis can result in DIIHA secondary to ceftriaxone even with a negative DCT [8].

Prompt withdrawal of the responsible drug is the most important therapeutic measure in order to stop further complement activation [2,9]. IVIG therapy is an option in severe cases, however, there is no proven benefit with steroid therapy [2,3,9]. The benefit of steroids and IVIG is confounded by simultaneous discontinuation of the inciting agent [2,3]. Plasmapheresis can also be used to filter out the remaining drug-induced antibodies and expedite the excretion of the remaining drug from the patient’s serum [2,10]. The drug should then be added to the patient’s medical record in order to prevent reexposure and cross-reactivity with other drugs of the same class [3,4,7].

## Conclusions

Ceftriaxone is a broad-spectrum antibiotic that can cause DIIHA in rare instances. Laboratory investigations usually identify hemolysis by decreased hemoglobin, low haptoglobin, elevated LDH, hyperbilirubinemia, and positive direct Coombs test for anti-C3 and, in some cases, IgG/IgM. Once DIIHA is confirmed, it is important to immediately discontinue ceftriaxone. A decrease in hemoglobin during ceftriaxone therapy should trigger the timely recognition of DIIHA in order to prevent worse outcomes for something with a simple treatment.
